# Impact of big data analytics on sales performance in pharmaceutical organizations: The role of customer relationship management capabilities

**DOI:** 10.1371/journal.pone.0250229

**Published:** 2021-04-28

**Authors:** Muhammad Shahbaz, Changyuan Gao, Lili Zhai, Fakhar Shahzad, Adeel Luqman, Rimsha Zahid

**Affiliations:** 1 School of Economics and Management, Harbin University of Science and Technology, Harbin, Heilongjiang, China; 2 Lyallpur Business School, Government Collage University, Faisalabad, Punjab, Pakistan; 3 Department of Business Administration, ILMA University, Karachi, Pakistan; 4 Department of Commerce, Fatima Jinnah Women University, Rawalpindi, Pakistan; King Abdulaziz University, SAUDI ARABIA

## Abstract

In this era of technology development, every business wants to equip its salesforce with a sustainable salesforce automation system to improve sales performance and customer relationship management (CRM) capabilities. This study investigates the impact of big data analytics (BDA) on CRM capabilities and the sales performance of pharmaceutical organizations. A research model was tested based on 416 valid responses collected from pharmaceutical companies through a structured questionnaire. Structural equation modeling (SEM) was employed using Smart-PLS3 to confirm the contribution of BDA to improving CRM capabilities and sales performance. The study finds that individual characteristics such as self-efficacy, playfulness, and social norms, along with organizational characteristics such as voluntariness, user involvement, user participation, and management support, are positive predictors of salesforce perception of BDA. This positive perception of BDA increased the person-technology fit in the salesforce, which ultimately increased the CRM capabilities and sales performance.

## Introduction

Current advances in information technology (IT) and the rising trend of social media have changed the way salespersons perform daily routine activities. Most often, the salesforce is equipped with a salesforce automation (SFA) system to enhance customer relationship management (CRM) capabilities and sales performance [1]. SFA systems are a set of tools that facilitate organization by providing analyzed information from available data to manage customer relationships and sales-related activities [2]. An SFA system provides information regarding customer interactions, inventory control, sales forecasting, sales, communication history, and pipeline opportunities to efficiently achieve day-to-day goals [3]. Organizations annually invest millions of dollars in the implementation of SFA systems to achieve excellent customer relations and sales progress [4]. However, the literature reports that overall, more than 61% of SFA systems fail to meet the current requirements of salesforces [3, 5–8]. The major causes of SFA system failure reported in the literature are ignoring important sources of data and inability to analyze the massive volume of data [7–9].

In the recent past, data about sales and CRM have been generated at a very high pace. Traditional systems are unable to store and analyze such high volume, variety, and velocity data (i.e., big data) to obtain information for decision making [10]. In this era of the big data (BD) revolution, the method for formulating strategy in sales and CRM has changed, and organizations should employ a data analytics system to fulfill the requirements of optimistic strategy formulation [11]. BD provides behavioral insights for organizations and customers, and analytics is used to extract fruitful information from BD for decision making. Big data analytics (BDA) is defined in this study as a technology or system that provides useful information about the behavior of customers by exploring hidden patterns in BD to support effective strategy-making for sales and CRM. Data related to customer buying behavior are being generated at unprecedented speeds due to the technology revolution and the advent of sources such as social media [12, 13]. The sources of data inputs in traditional sales support systems (i.e., SFA systems) are boundary spanners [7, 8], and the input data only represent the organization [14]. This limitation is one of the primary reasons for the failure of these systems, as they ignore important data relevant to sales and customers from social media and other key sources and ignore the data of competitor organizations. In this modern era, data from social media are one of the most important sources for predicting customer behavior regarding buying preferences [9, 15], and competitor and market stakeholder information can play a significant role in developing strategies [16, 17]. The inability to store and analyze the newly generated and existing uncollected massive volume and variety of data is another reason for the failure of SFA systems [18]. However, BDA extracts customer opinions on products, services, and organizations by mining customer data from all possible sources, e.g., social media data, sensor network data, transactional data, and survey data, for decision making and has the ability to analyze massive amounts and varieties of data [19, 20]. Therefore, based on prior literature, this study recognizes that BDA can overcome some shortcomings of SFA, which is the motivation to do this research. To fill this research gap, the main objective of this study is to present BDA as an alternative to SFA to increase sales performance and CRM capabilities. Therefore, keeping this objective in mind, we propose a theory-driven research model to investigate the impact of BDA on sales performance and CRM capabilities, which has not been investigated by previous scholars in this study context.

In this study, the individual means employees of the organization and these are the most important entity in the context of big data analytics because employees are responsible for dealing with BDA at the organizational level, e.g. hardware, software, and other technical aspects. Employees are key stakeholders in the organization, they not only use the output of the big data analytics but also need to operate BDA to produce valuable results for customers and end users. Several studies from the literature [21–25] identify the importance of employees’ perception of BDA towards successful implementation of it and strong impact on different level organizational performances including sales performance. Therefore, to study different factors that build employees’ perception of BDA and create the sense of person technology fit in this study context is the need of time.

Most of the literature highlights the rejection of SFA by several organizations because a positive perception about the system has not been created or maintained due to its deficiencies, and ultimately, the negative perception creates a belief in a job and professional misfit between the system and the salesforce [3, 6, 7]. The identity theory demonstrates that a positive perception of technology creates the perception of job and profession fit for employees, which ultimately becomes the reason for increases in sale performance and CRM capabilities and decreases in employee turnover [26–28]. Therefore, this study proposes a research model to fulfill the objective of this study by examining how individual characteristics combined with organizational characteristics and along with innovation diffusion theory (IDT) can build a positive perception of BDA in the employees of organizations. Moreover, this study uses the identity theory to describe how the positive perception of BDA creates a technology fit perception among employees of sales department to ultimately impact the organization’s sales performance and CRM capabilities.

## Development of hypotheses and research model

### Individual characteristics

In this study context, individual characteristics mean the characteristics of organization’s employees. An individual’s beliefs affect the association between external characteristics and consequences [29]. A person’s characteristics can direct his perception of a specific system and benefit from the system. In this study, individual characteristics are those characteristics that relate to the perception of BDA and its expected outcomes and that aid in adopting BDA. In this research, we examined self-efficacy, playfulness, and social norms as individual factors. The theory of self-efficacy already established the strong relationship of self-efficacy and playfulness with the user perception or positive intention [30]. Moreover, theory of planned behavior already proved theoretical support in the strong relationship of social norms and individual perception of user about the system [31]. Computer self-efficiency is the degree of ability that makes the employees capable of finishing a specific task by using technology or a system [6], for example, using BDA to perform a task related to sales and customer relations. Only a few salespeople have the ability to use technology, and the remainder have just a little bit of knowledge about the system [32, 33]. A low level of computer self-efficacy is one of the reasons for negative perception among employees about the system [34, 35]. Computer playfulness is an individual’s propensity to respond immediately to computer-related tasks. Computer playfulness has the potential to affect technology acceptance and perception [6]. Playfulness is about the spirit to use the computer-based technologies and BDA is also a technology that based on computer. It means to run BDA, employees of organization have the spirit and skills to use computer. Prior studies and theories already concluded that playfulness of user has strong influence on many complex computer based technologies [36–38]. Therefore, it is significant to study the impact of playfulness in this study context. It is evident from the previous literature that people who have inherent computer playfulness characteristics are always passionate about using a system, which ultimately leads towards the positive perception of the system [39, 40]. Social norms often refer to how employee perceptions change towards technological system acceptance in light of the social group’s response [41]. Expected social loss prevents an employee from adopting a technological system [42]. Social norms or subjective norms are a key factor in determining the acceptance and perception of a system [34, 43]. As manifested in the previous literature, these factors have a significant influence on the perception of different systems in different sectors [6, 40, 42]. Therefore, this study hypothesized that these factors would influence the individual perception of BDA, as follows:

**H1:** Individual characteristics (self-efficacy, playfulness, and social norms) are positively associated with the individual’s perception of BDA.

### Organizational characteristics

Previous studies highlighted some organizational factors that have a significant impact on the perception of the system [6, 44–46], and these factors are also expected to influence BDA acceptance. In this study, an organizations characteristics means all those employee level factors that organizations can create or improve in employees and these factors ultimately have strong impact on the employee perception. User participation is the contribution and performance of employees or their representatives in the system development process [47], while user involvement is the psychological state of the user that defines his relevance to the system [47]. The behavioral theory of information system success and technology acceptance model 2 already established the strong impact of voluntariness, user involvement, user participation and management support on the perception of employee or user of the system [48, 49]. Prior studies proved that user participation and user involvement have a significant relationship with the individual perception of a system [6, 47]. Therefore, we consider that user participation and involvement would also have an impact on an individual’s perception of BDA. Management support refers to top management participation and involvement in the process of system development and implementation in regard to supporting the user’s adoption of the system. Management support also has a significant influence on the perception of the system [50]. Voluntariness is the extent to which the use of the system is perceived to be not required by the organization [51, 52]. Past literature has established that the extent to which adoption of a system is perceived as voluntary has positive consequences on the perception of the system that ultimately leads towards its acceptance [47]. Therefore, we also consider that the above discussed organizational characteristics can influence the individual’s perception of BDA towards acceptance.

**H2:** Organizational characteristics (voluntariness, user involvement, user participation, and management support) are positively associated with the individual perception of BDA.

### Individual perception of BDA

Prior studies by Moore and Rogers [52, 53] explored the user’s acceptance of the technological system by demonstrating innovation diffusion theory. A positive employee perception of any system ultimately leads the employee towards its adoption and use. In this study the individual refers to the employees of the organization. Relative advantage, visibility, image, compatibility, complexity, and result demonstration are the factors that lead the employees to perceive the system positively and consider its adoption [52]. Relative advantage is the degree to which a system is perceived as more effective than its forerunner. Compatibility is the degree to which a system is well-matched with existing norms, beliefs, values, and preceding experience. Complexity can be defined as the degree to which an employee of an organization perceives that the use of the system will require his or her mental and physical effort. Visibility is how a system is perceived or seen in the organization. Image is an employee’s perception about a system in terms of how much using the system will enhance his status in the social system. Results demonstration is the ability of the system to display benefits that are visible.

The theory of social identity concluded that individual positive perception of the system or technology user create the positive perception about the job and profession fit that ultimately increase the performance of the organizations [54]. Furthermore, the task-technology fit theory also justify this logic that different task characteristics and technology characteristics make the positive perception in user about the fitness of the job with the technology and ultimately it increases the performance [55]. Therefore, in this study the authors also proposed the relationship of Individual perception of BDA and person-technology fit based on prior theories. It is evident from the literature that positive perceptions of different systems create a sense of fit between the job, professional requirements, and the system [51]. Furthermore, the job and professional perceived requirements are associated with the individual’s perception of the system [56, 57]. In this study, we consider that the individual’s perception of BDA will lead the salesforce towards a person-technology fit belief. It is also proven from previous studies that a positive individual perception of the system creates a positive belief in salespersons that the system can fulfill the job and professionally related expectations [6]. Therefore, we hypothesized the following:

**H3:** Individual perception of BDA (relative advantage, visibility, image, compatibility, complexity and result demonstrability) are positively associated with the person-technology fit.

### Person-technology fit

The social identity theory sheds light on the different roles that the employee performs in personal and professional life [58]. The model suggests that employees build up expectations and hopes through specific roles and act to maintain those expectations and hopes [59]. An individual, in particular, sees himself in different roles [54], e.g., a salesperson would consider him or herself in two different roles: a professional identity and an organizational identity. In the professional identity, he or she is a salesperson, and in the organizational identity, he or she is the manager of a specific territory for his or her organization. Occasionally, these roles are in conflict with each other, and an activity executed in a specific role is inconsistent with the expectation of another role. There could be an important conflict between the expectations of the two roles.

The question is how a specific system can affect these roles. It is evident from the previous literature that a specific system can increase or decrease competency depending upon the individual’s socially constructed expectations [60]. The system will increase competency when it builds up and improves the level of current skills, relationships, and knowledge The system will decrease competency when it decreases or has no impact on current skills, relationships or knowledge [6], and it creates a positive or negative perception of the job [61]. By using BDA in sales, there is a heavy burden of routine tasks such as receiving and replying to emails and electronically interacting with coworkers. There is less time to meet face-to-face with organizational coworkers and customers [62], which might lead to a negative perception of the system. Hence, it is important that salespeople have a positive perception of the system before use [6] and analyze how BDA is consistent with their job and profession. Being consistent with job and profession reflects the degree to which BDA increases job and professional competencies in regard to long-term career prospects.

Therefore, a positive perception of person-technology fit will increase the socially constructed expectations and hopes that ultimately bring positive outcomes in terms of adoption of the system and will increase employee performance in sales and CRM capabilities; in contrast, a negative perception of person-technology fit brings negative outcomes. Therefore, this study proposes the following:

**H4:** Person-technology fit (job fit and profession fit) is positively associated with CRM capabilities.
**H5:** Person-technology fit (job fit and profession fit) is positively associated with sales performance.

### CRM capabilities

CRM is a cross-functional mechanism by which organizations create, maintain, and strengthen a long-lasting relationship with the customer [63–65]. CRM capabilities strategically link information technology and marketing strategies for long-term customer relationships [66]. The success of CRM capabilities depends upon the data and analytics that are being used. CRM is an essential part of the success of every organization and has many capabilities, including customer knowledge capabilities, information infrastructure capabilities, customer strategy capabilities and structure capabilities [67]. The key elements that enhance CRM capabilities are data collection and analytics systems [68]. In this study, we believe that BDA will increase CRM capabilities.

In addition, CRM capabilities help strategy makers and salespeople increase sales performance [22, 69]. The common guarantee of CRM capabilities is that they make sales procedures more effective and efficient and thereby increase sales performance [70]. CRM capabilities provide assistance to the sales team to specifically target relevant customers and not waste time on irrelevant customers to reduce the sales cycle time and cost [71]. Therefore, in light of previous literature, we also hypothesized the following:

**H6:** CRM capabilities are positively associated with sales performance.

### Sales performance

Sales performance effectively and efficiently achieves the targets in the sales process by examining opportunities and improving closing rates [72]. The information technology system (i.e., BDA) has been observed to aid salespersons in obtaining better closing rates and increasing revenue [73]. The salesperson can increase their knowledge, targeting, and presentation skills by taking advantage of information technology system capabilities, i.e., BDA capabilities [74]. In the current study, we consider that BDA will enhance sales performance.

**Fig 1:**
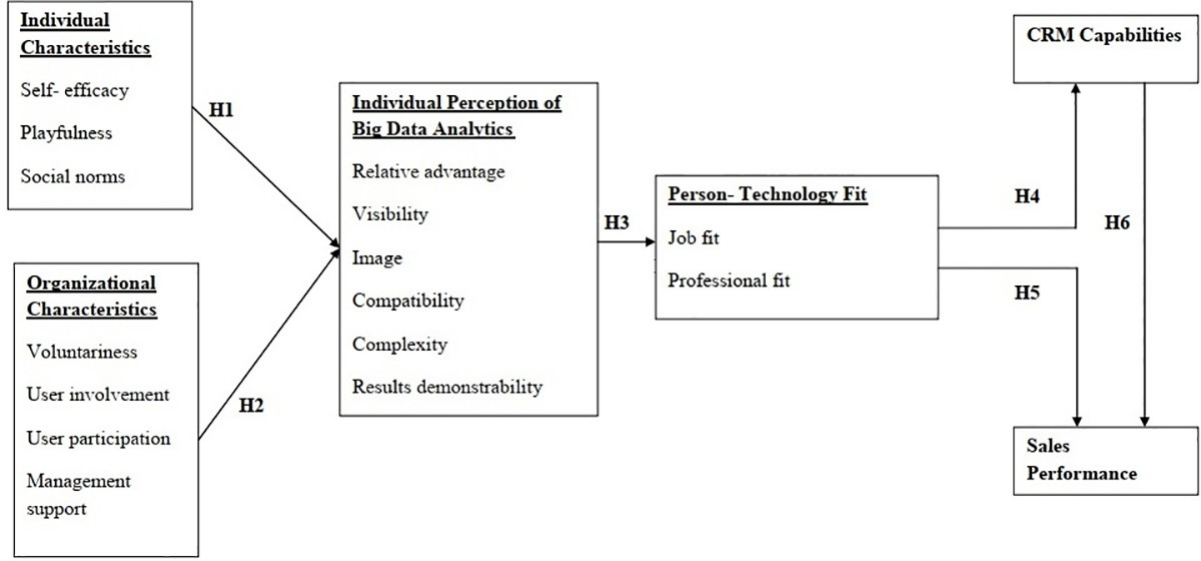
Proposed research model.

A theoretical model that organizes this study is presented in Fig 1; bold text represents the basic research framework, and the constructs under the bold text are operational constructs.

## Materials and methods

### Measures

We adapted measures from previous studies that are appropriate in this study context. The reflective nature of scales used in this study. **Table A1** in S1 Appendix is presenting the details of measurement items. The eight-item scale of self-efficacy was adapted from [75], the seven-item scale of playfulness was adapted from [76], the four-item social norms scale was adapted from [41], and the four-item scale of voluntariness was adapted from [52]. Similarly, the three-item scale of user involvement was adapted from [47] and that for user participation from [47]. The three-item scale of management support was adapted from [77]. The three-item scale of relative advantage, visibility, image, compatibility, and result demonstrability and the four-item scale of complexity were adapted from [52]. The five-item scale of job fit and the six-item scale of profession fit were adapted from [56]. Furthermore, the three-item scale of CRM capabilities was adapted from [67] and that for sales performance from [78].

The final questionnaire items were measured on a seven-point Likert scale ranging from 1 (strongly disagree) to 7 (strongly agree). The seven-point Likert scale is the most important and widely used scale in survey instruments [79, 80]. Furthermore, to ensure the validity of the questionnaire,40 participants from the target population who were not included in the study analysis were used in a pilot study. After the pilot test questionnaire was discussed, three professors who are experts in survey instruments were asked to further refine the questionnaire. The pilot testing results of the questionnaire are presented in **Table A2** in S1 Appendix. The results verified the validity of the instrument, as the Cronbach’s alpha scores for all the factors ranged from 0.788 to 0.969, which are above the threshold value. Moreover, there is no factor loading problem in the factors of the instrument.

### Sampling and data collection method

This study is related to the quantitative research method. Therefore, a survey was conducted to examine the hypotheses and the relationships among constructs [81, 82].We selected the sales managers of pharmaceutical companies operating in Pakistan, who are the true representatives of the salesforce team of the organization. The pharmaceutical sector is rapidly growing and an important contributor to the economy of Pakistan [83] but faces the problem of timely decision making due to the lack of a proper information system [84, 85]. Data regarding the number of pharmaceutical organizations operating in Pakistan and their contact information have been taken from the Drug Regulatory Authority of Pakistan (DRAP), a regulatory body that controls the pharmaceutical sector [86]. The human resource departments (HRDs) of the pharmaceutical organizations were contacted, and with the help and permission of the HRDs, the data were collected from the sales managers for the present study. We ensured the organizations that the data collected from employees would be used only for research purposes and that we would share the findings of the research with them. An online survey used to complete the questionnaire. A total of 800 questionnaires were distributed, of which 416 were selected for analyses after those with missing values and biased responses were discarded. The study used G*Power software to predict a suitable sample size, as it is a powerful tool for ensuring sample adequacy before and after data collection [87].While applying G*Power, the total required sample size was 262 for “A priori: Compute required sample size”. Meanwhile, applying “Post hoc: Compute achieved power” on the collected sample size of 416 determined the power to be 0.995, showing the adequate strength of the study sample.

### Demographics of respondents

The demographics of participants are displayed in **Table 1** and describethat77.9% of the participants are male, and 63.7% are between the ages of 30–39 years. The majority of participants are highly educated, i.e., 31.5% are graduates, and 21.6% are postgraduates. Therefore, our participants were young and highly educated. Furthermore, the study also considered gender, age and education as control variables and all the control variables are insignificant.

**Table 1:** Demographic variables.

Category		Frequency	Percentage
**Gender**	Male	324	77.9
	Female	92	22.1
	Total	416	100.0
**Age**	18–29	98	23.6
	30–39	265	63.7
	40–50	34	8.2
	Above 50	19	4.6
	Total	416	100.0
**Education**	Undergraduate	73	17.5
	Graduate	131	31.5
	Postgraduate	90	21.6
	Other (Diploma/ Professional education)	122	29.3
	Total	416	100.0

### Common method bias

Common method bias (CMB) is a serious issue to be addressed if the data are gathered from a single source and at the same point of time [88]. Therefore, we used Harman’s single factor test to ensure that there was no CMB. The results divided the factors into seventeen, and the first factor explained 37.9% of the variance, which is below the threshold of 40% [89]. Only Harman’s single factor test is not sufficient to test the CMB. Therefore, this study also applied Inner variance inflation factor (VIF) to address the CMB. The values of inner VIF should be below 3.3 [90].The inner values of this study are ranging from 1.402 to 2.096 which are below the threshold value. The above results proved that CMB is not an issue in this study.

## Results

Partial least squares-structural equation modeling (PLS-SEM) was applied to test the hypotheses by means of Smart-PLS v3. PLS-SEM is an important analysis technique to test the theoretical framework from the perspective of prediction [91]. As studies confirmed that a consistent PLS algorithm allows for the adjustment of the reflective construct correlations [92, 93]. Therefore, we used a consistent PLS algorithm in this study. The results from measurement and structural model are given below.

### Measurement model

The study followed the method proposed by [94] to evaluate the measurement model by measuring the content, convergent, and discriminant validities. The content validity is achieved by reviewing the relevant literature and the pilot study of the instrument. Convergent validity was achieved by examining factor loading, Cronbach’s alpha, composite reliability (CR), and the average variance extracted (AVE).

**Table 2** describes the results and shows that the factor loadings of all items are above 0.7, which is acceptable. The threshold values of Cronbach’s alpha, AVE, and CR are 0.70, 0.50, and 0.70, respectively [95]. All the values of Cronbach’s alpha, AVE, and CR are above the cutoffs. Therefore, there is no issue of convergent validity.

**Table 2:** Results of factor loadings, Cronbach’s alpha, CR, and AVE.

Constructs	Items	Loadings	Cronbach’s Alpha	CR	AVE
**Self- efficacy**	SE1	0.860	0.960	0.960	0.749
	SE2	0.875			
	SE3	0.848			
	SE4	0.925			
	SE5	0.905			
	SE6	0.841			
	SE7	0.851			
	SE8	0.811			
**Playfulness**	P1	0.912	0.967	0.967	0.806
	P2	0.837			
	P3	0.965			
	P4	0.858			
	P5	0.957			
	P6	0.847			
	P7	0.898			
**Social Norms**	SN1	0.924	0.959	0.960	0.856
	SN2	0.973			
	SN3	0.891			
	SN4	0.912			
**Voluntariness**	VN1	0.764	0.899	0.899	0.691
	VN2	0.862			
	VN3	0.828			
	VN4	0.868			
**User involvement**	UI1	0.794	0.873	0.873	0.697
	UI2	0.842			
	UI3	0.867			
**User participation**	UP1	0.813	0.850	0.851	0.656
	UP2	0.852			
	UP3	0.762			
**Management support**	MS1	0.769	0.856	0.857	0.666
	MS2	0.866			
	MS3	0.810			
**Relative advantage**	RAD1	0.743	0.795	0.795	0.565
	RAD2	0.803			
	RAD3	0.705			
**Visibility**	V1	0.909	0.972	0.972	0.922
	V2	0.990			
	V3	0.980			
**Image**	I1	0.853	0.927	0.929	0.814
	I2	0.875			
	I3	0.973			
**Compatibility**	C1	0.859	0.916	0.916	0.785
	C2	0.873			
	C3	0.924			
**Complexity**	CLX1	0.941	0.967	0.967	0.880
	CLX2	0.938			
	CLX3	0.950			
	CLX4	0.923			
**Results demonstrability**	RD1	0.854	0.861	0.861	0.674
	RD2	0.831			
	RD3	0.775			
**Job Fit**	JF1	0.817	0.917	0.917	0.690
	JF2	0.802			
	JF3	0.785			
	JF4	0.871			
	JF5	0.874			
**Professional Fit**	PF1	0.846	0.911	0.911	0.630
	PF2	0.803			
	PF3	0.798			
	PF4	0.767			
	PF5	0.777			
	PF6	0.770			
**CRM capabilities**	CRM1	0.866	0.884	0.885	0.720
	CRM2	0.881			
	CRM3	0.795			
**Sales Performance**	SP1	0.830	0.841	0.841	0.639
	SP2	0.799			
	SP3	0.768			

Discriminant validity was achieved by using three techniques as suggested by [96]. The first technique was the association between the correlations of factors and the square root of AVE, as described by [97], which is the best technique for measuring discriminant validity. The second technique was to probe the item loadings and cross-loadings to certify the correlations and the third one was applying the Heterotrait-Monotrait Ratio (HTMT) [98, 99] **Table 3** shows that the values of the square root of AVE are greater than the coefficients of correlation of all variables with each other. Second, the cross-loadings and item loadings of all corresponding variables are higher than the cross-loadings and item loadings of other latent variables that proves that there is no issue regarding discriminant validity. The HTMT ratio criterion was established to specify insensitivity of cross loadings technique and Fornell-Larcker technique. The HTMT is estimation of factors correlation and in other words the upper boundary. HTMT should be less than 1 to clearly distinguish two factors [99]. **Table 4** described that highest value is 0.665 that is under the threshold value and confirms the no discriminant validity issue in this study. All the results illustrate that no issue of content, convergence, or discriminant validity existed in this study and grants the go-ahead to use the data for the structural model.

**Table 3:** Inter-construct correlations and discriminant validity.

	RAD	CLX	CRM	I	JF	MS	P	PF	C	RD	SE	SN	SP	UI	UP	V	VN
**RAD**	**0.752**																
**CLX**	0.485	**0.938**															
**CRM**	0.442	0.522	**0.848**														
**I**	0.476	0.573	0.543	**0.902**													
**JF**	0.561	0.581	0.537	0.520	**0.831**												
**MS**	0.428	0.454	0.455	0.491	0.465	**0.816**											
**P**	0.412	0.444	0.366	0.385	0.411	0.359	**0.898**										
**PF**	0.535	0.611	0.545	0.616	0.536	0.432	0.414	**0.794**									
**C**	0.474	0.605	0.523	0.606	0.563	0.426	0.405	0.665	**0.886**								
**RD**	0.500	0.489	0.416	0.551	0.537	0.426	0.428	0.509	0.534	**0.821**							
**SE**	0.435	0.477	0.381	0.484	0.495	0.439	0.437	0.434	0.446	0.522	**0.865**						
**SN**	0.477	0.518	0.453	0.494	0.500	0.412	0.453	0.465	0.453	0.397	0.429	**0.925**					
**SP**	0.434	0.455	0.561	0.489	0.575	0.506	0.356	0.483	0.453	0.479	0.419	0.425	**0.799**				
**UI**	0.475	0.443	0.457	0.460	0.472	0.452	0.391	0.376	0.473	0.457	0.424	0.504	0.410	**0.835**			
**UP**	0.427	0.501	0.461	0.448	0.491	0.496	0.406	0.480	0.466	0.447	0.440	0.465	0.450	0.435	**0.810**		
**V**	0.484	0.526	0.423	0.507	0.642	0.433	0.419	0.557	0.557	0.489	0.444	0.401	0.454	0.379	0.457	**0.960**	
**VN**	0.413	0.419	0.506	0.486	0.51	0.433	0.345	0.483	0.514	0.446	0.437	0.497	0.549	0.498	0.437	0.435	**0.831**

**Table 4:** HTMT ratio criterion.

	C	CLX	CRM	I	JF	MS	P	PF	RAD	RD	SE	SN	SP	UI	UP	V
CLX	0.604															
CRM	0.522	0.522														
I	0.608	0.574	0.545													
JF	0.564	0.580	0.537	0.521												
MS	0.424	0.453	0.455	0.491	0.465											
P	0.404	0.443	0.364	0.385	0.408	0.357										
PF	0.665	0.611	0.544	0.618	0.534	0.432	0.413									
RAD	0.473	0.484	0.440	0.476	0.562	0.430	0.411	0.535								
RD	0.534	0.487	0.415	0.551	0.536	0.426	0.425	0.508	0.500							
SE	0.445	0.476	0.381	0.484	0.494	0.438	0.436	0.432	0.433	0.520						
SN	0.452	0.518	0.453	0.495	0.499	0.410	0.452	0.466	0.477	0.397	0.428					
SP	0.451	0.456	0.560	0.490	0.574	0.508	0.355	0.482	0.436	0.480	0.420	0.425				
UI	0.472	0.442	0.458	0.460	0.471	0.451	0.390	0.375	0.476	0.457	0.423	0.505	0.409			
UP	0.467	0.501	0.460	0.448	0.492	0.498	0.405	0.481	0.424	0.447	0.439	0.467	0.453	0.435		
V	0.557	0.527	0.422	0.507	0.643	0.433	0.417	0.556	0.484	0.489	0.443	0.401	0.454	0.379	0.458	
VN	0.513	0.418	0.506	0.486	0.509	0.432	0.345	0.483	0.413	0.445	0.436	0.496	0.548	0.496	0.437	0.436

### Structural model

Smart PLS3 was used to test the relationship among constructs based on standardized paths [92]. **Table 5** presents the path coefficient, t-values, and p-values. The results in **Table 6** show that the model explains 43.1% of the variance in sales performance, 38.1% of the variance in CRM capabilities, 57.9% of the variance in profession fit, 55.9% of the variance in job fit, and 37.6%, 35.9%, 42.5%, 40.8%, 42.9%, and 40.4% of the variance in relative advantage, visibility, image, compatibility, complexity, and results demonstrability, respectively. Table 4 show that individual characteristics, i.e., self-efficacy, playfulness, and social norms, have a significant relationship with all six constructs of the individual perception of BDA (relative advantage, visibility, image, compatibility, complexity, and results demonstrability) except the relationship between H1_i_: playfulness and image, H1_n_: social norms and visibility, H1_p_: social norms and compatibility, and H1_r_: social norms & result demonstrability. Our results are consistent with the findings of [6, 34], as self-efficacy (β = 0.156, p = 0.003), playfulness (β = 0.127, p = 0.015), and social norms (β = 0.206, p = 0.000) have a significant positive relationship with complexity. Therefore, we accepted H1. Furthermore, H2 is partially supported because fifteen out of twenty-four relationships have a significant positive relationship. The finding that not all the factors of organizational characteristics influenced all the factors of individual perception of BDA is consistent with previous studies [6, 51]. This study’s results regarding hypothesis 2 are consistent with the studies of Speier and Agarwal [6, 51]. Therefore, this study accepted H2. H3 is also partially supported because nine out of twelve relationships between factors of individual perception of BDA and the factors of person-technology fit are positively associated. The results of the study are also consistent with the findings of Speier [6] as relative advantage (β = 0.148, p = 0.008 & β = 0.195, p = 0.000) has a positively significant relationship with profession fit and job fit, respectively. Therefore, H3 was also accepted in this study. Moreover, job fit (β = 0.343, p = 0.000 and β = 0.336, p = 0.000) has a significant positive relationship with CRM capabilities and sales performance, respectively. Similarly, profession fit (β = 0.361, p = 0.000 and β = 0.136, p = 0.039) also has a significant positive relationship with CRM capabilities and sales performance, respectively. CRM capabilities (β = 0.306, p = 0.000) also have a significant positive relationship with sales performance, and these results are consistent with the study of Rodriguez [72]. On the basis of all results, we accept our proposed research model.

**Table 5:** SEM hypotheses results.

Hypotheses				Path coefficient	t-value	p-value	Results
H1	**Individual characteristics ➝ Individual perception of big data analytics**						Supported
H1a	Self- efficacy	**➝**	Relative advantage	0.123	2.065	0.039	Supported
H1b	Self- efficacy	**➝**	Visibility	0.148	2.796	0.005	Supported
H1c	Self- efficacy	**➝**	Image	0.171	3.226	0.001	Supported
H1d	Self- efficacy	**➝**	Compatibility	0.115	2.134	0.033	Supported
H1e	Self- efficacy	**➝**	Complexity	0.156	2.970	0.003	Supported
H1f	Self- efficacy	**➝**	Result demonstrability	0.256	4.708	0.000	Supported
H1g	Playfulness	**➝**	Relative advantage	0.117	1.922	0.050	Supported
H1h	Playfulness	**➝**	Visibility	0.156	3.207	0.001	Supported
H1i	Playfulness	**➝**	Image	0.050	0.955	0.340	Not Supported
H1j	Playfulness	**➝**	Compatibility	0.103	1.998	0.046	Supported
H1k	Playfulness	**➝**	Complexity	0.127	2.434	0.015	Supported
H1l	Playfulness	**➝**	Result demonstrability	0.140	2.539	0.011	Supported
H1m	Social Norms	**➝**	Relative advantage	0.165	2.598	0.009	Supported
H1n	Social Norms	**➝**	Visibility	0.049	0.910	0.363	Not Supported
H1o	Social Norms	**➝**	Image	0.163	2.876	0.004	Supported
H1p	Social Norms	**➝**	Compatibility	0.078	1.372	0.170	Not Supported
H1q	Social Norms	**➝**	Complexity	0.206	4.002	0.000	Supported
H1r	Social Norms	**➝**	Result demonstrability	0.000	0.007	0.994	Not Supported
H2	**Organizational characteristics ➝ Individual perception of big data analytics**						Supported
H2a	Voluntariness	**➝**	Relative advantage	0.062	0.889	0.374	Not Supported
H2b	Voluntariness	**➝**	Visibility	0.151	2.825	0.005	Supported
H2c	Voluntariness	**➝**	Image	0.153	2.771	0.006	Supported
H2d	Voluntariness	**➝**	Compatibility	0.229	4.098	0.000	Supported
H2e	Voluntariness	**➝**	Complexity	0.040	0.719	0.472	Not Supported
H2f	Voluntariness	**➝**	Result demonstrability	0.127	2.181	0.029	Supported
H2g	User involvement	**➝**	Relative advantage	0.173	2.708	0.007	Supported
H2h	User involvement	**➝**	Visibility	0.023	0.393	0.694	Not Supported
H2i	User involvement	**➝**	Image	0.092	1.490	0.136	Not Supported
H2j	User involvement	**➝**	Compatibility	0.135	2.309	0.021	Supported
H2k	User involvement	**➝**	Complexity	0.073	1.324	0.186	Not Supported
H2l	User involvement	**➝**	Result demonstrability	0.141	2.161	0.031	Supported
H2m	User participation	**➝**	Relative advantage	0.089	1.492	0.136	Not Supported
H2n	User participation	**➝**	Visibility	0.162	2.658	0.008	Supported
H2o	User participation	**➝**	Image	0.078	1.374	0.170	Not Supported
H2p	User participation	**➝**	Compatibility	0.141	2.403	0.016	Supported
H2q	User participation	**➝**	Complexity	0.178	3.433	0.001	Supported
H2r	User participation	**➝**	Result demonstrability	0.119	2.017	0.044	Supported
H2s	Management support	**➝**	Relative advantage	0.117	1.891	0.050	Supported
H2t	Management support	**➝**	Visibility	0.137	2.402	0.016	Supported
H2u	Management support	**➝**	Image	0.185	3.349	0.001	Supported
H2v	Management support	**➝**	Compatibility	0.076	1.396	0.163	Not Supported
H2w	Management support	**➝**	Complexity	0.118	2.181	0.029	Supported
H2x	Management support	**➝**	Result demonstrability	0.087	1.439	0.150	Not Supported
H3	**Individual perception of BDA ➝ Person-technology fit**						Supported
H3a	Relative advantage	**➝**	Job Fit	0.195	3.859	0.000	Supported
H3b	Visibility	**➝**	Job Fit	0.323	6.569	0.000	Supported
H3c	Image	**➝**	Job Fit	0.036	0.668	0.491	Not Supported
H3d	Compatibility	**➝**	Job Fit	0.095	1.726	0.085	Not Supported
H3e	Complexity	**➝**	Job Fit	0.177	3.620	0.000	Supported
H3f	Result demonstrability	**➝**	Job Fit	0.124	2.144	0.032	Supported
H3g	Relative advantage	**➝**	Professional Fit	0.148	2.644	0.008	Supported
H3h	Visibility	**➝**	Professional Fit	0.118	2.282	0.023	Supported
H3i	Image	**➝**	Professional Fit	0.191	3.318	0.001	Supported
H3j	Compatibility	**➝**	Professional Fit	0.289	5.145	0.000	Supported
H3k	Complexity	**➝**	Professional Fit	0.178	3.396	0.001	Supported
H3l	Result demonstrability	**➝**	Professional Fit	0.031	0.585	0.559	Not Supported
H4	**Person-technology fit ➝ CRM**						Supported
H4a	Job Fit	**➝**	CRM capabilities	0.343	6.170	0.000	Supported
H4b	Professional Fit	**➝**	CRM capabilities	0.361	6.257	0.000	Supported
H5	**Person-technology fit ➝ Sales performance**						Supported
H5a	Job Fit	**➝**	Job Fit performance	0.336	5.719	0.000	Supported
H5b	Professional Fit	**➝**	Professional Fit Sales performance	0.136	2.064	0.039	Supported
H6	**CRM capabilities**	**➝**	**Sales performance**	0.306	4.718	0.000	Supported

**Table 6:** Values of R- square.

Variables	Values of R- Square
Sales performance	**0.431**
CRM Capabilities	**0.381**
Professional fit	**0.579**
Job fit	**0.559**
Relative advantage	**0.376**
Visibility	**0.359**
Image	**0.425**
Compatibility	**0.408**
Complexity	**0.429**
Results demonstrability	**0.404**

Moreover, the study conducted the PLS predict test to confirm the predictive accuracy of the model. The data of the present study is symmetrical distributed so we considered RMSE values. **Table 7** presented the results of PLS predict test. According to the results all the values of Q^2^_predict are above zero that shows there is nothing wrong with it. Furthermore, mostly the differences between the values of PLS-RMSE and ML-RMSE are below zero that concluded that the research model of the present study have the moderate predictive power [100].

**Table 7:** Results of PLS predict test.

	PLS-RMSE	Q^2^_predict	ML-RMSE	Difference		PLS-RMSE	Q^2^_predict	ML-RMSE	Difference
**C3**	0.702	0.314	0.736	-0.033	**PF2**	0.789	0.228	0.822	-0.034
**C1**	0.722	0.253	0.753	-0.031	**PF4**	0.803	0.213	0.826	-0.023
**C2**	0.683	0.274	0.720	-0.038	**PF5**	0.838	0.222	0.875	-0.036
**CLX2**	0.629	0.345	0.660	-0.031	**PF1**	0.796	0.258	0.826	-0.030
**CLX1**	0.617	0.343	0.641	-0.023	**PF6**	0.839	0.204	0.884	-0.045
**CLX3**	0.615	0.356	0.643	-0.027	**PF3**	0.776	0.239	0.804	-0.028
**CLX4**	0.618	0.341	0.647	-0.029	**RAD1**	0.802	0.204	0.833	-0.030
**CRM1**	0.672	0.213	0.680	-0.009	**RAD2**	0.863	0.206	0.893	-0.030
**CRM3**	0.705	0.194	0.702	0.003	**RAD3**	0.860	0.156	0.883	-0.023
**CRM2**	0.662	0.228	0.640	0.022	**RD3**	0.708	0.194	0.733	-0.025
**I2**	0.712	0.295	0.745	-0.033	**RD1**	0.676	0.292	0.685	-0.009
**I3**	0.702	0.368	0.725	-0.024	**RD2**	0.674	0.248	0.708	-0.033
**I1**	0.739	0.279	0.772	-0.033	**SP2**	0.768	0.190	0.778	-0.011
**JF3**	0.717	0.244	0.740	-0.023	**SP1**	0.806	0.198	0.797	0.009
**JF5**	0.695	0.325	0.708	-0.013	**SP3**	0.792	0.190	0.782	0.010
**JF1**	0.719	0.275	0.744	-0.024	**V3**	0.670	0.312	0.681	-0.011
**JF4**	0.693	0.295	0.719	-0.026	**V2**	0.664	0.330	0.679	-0.016
**JF2**	0.703	0.267	0.718	-0.014	**V1**	0.687	0.259	0.701	-0.013

## Discussion

The present study results investigated specific concern of organizations regarding the integration of technology to strength the boundary spanning relationship of customers and sales managers. The overall results of this study proved that individual characteristics of employees of pharmaceutical organizations along with the organizational characteristics are strong influencers of Individual perception of BDA and measuring factors about individual perception of BDA is strong predictor of person technology fit. These results of the study are consistent with the general theories of technology acceptance [34, 101]. Furthermore, the results also verified the strong impact of person technology fit factors on CRM capabilities and sales performance and impact of CRM capabilities on sales performance as well.

According to the specific results of this study, all the relationships among individual characteristics (self-efficacy, playfulness, and social norms) and individual perception of BDA characteristics (relative advantage, image, visibility, compatibility, complexity, and result demonstrability) have significant relationships with each other except for the relationships between playfulness and image, social norms and visibility, social norms and compatibility, and social norms and results demonstrability. Self-efficacy is a strongest individual characteristic that influence the individual perception of BDA. Playfulness is not concerned with the image of the BDA because if playfulness increases, the ease of BDA in the mind of the user also increases, which means that playfulness does not have a significant relation with the image. The study of Jeffrey [37] also concluded that playfulness has an impact on the compatibility, complexity, and relative advantage of the system. Furthermore, the study of Speier &Venkatesh [14] concluded that social norms are a significant predictor of image because social norms can increase or decrease the image of any technology or system that further increases or decreases complexity in mind of the user and ultimately pulls users towards the adoption or rejection of technology. Therefore, our results are also consistent with the trend of prior literature in which social norms have a significant relationship with image and complexity [102].

Since self-efficacy is the most important predictor of individuals’ perception, organizations should pay more attention to self-efficacy. Playfulness and social norms are the second and third most important predictors of individual perception of BDA, respectively. The pharmaceutical organizations also consider that these factors help develop a better perception of BDA. Moreover, organizational characteristics (voluntariness, user involvement, user participation, and management support) and individual perception of BDA characteristics (relative advantage, image, visibility, compatibility, complexity, and result demonstrability) have significant relationships with each other, except for the relationships of voluntariness with relative advantage and complexity; user involvement with visibility, image, and complexity; user participation with relative advantage and image; and management support with compatibility and result demonstrability.

The perception is general, and this study also develops the same belief that voluntariness will increase the image and compatibility of BDA in the mind of the user; user involvement and user participation will also increase compatibility between the needs of the user and the BDA; and management support will significantly influence the visibility, image, and complexity of the BDA. These study results justify the study requirements, and the results are consistent with those of previous studies [6, 51], as previous studies concluded that every organizational characteristic should have a significant relationship with at least one factor of individual perception of technology or the system. The pharmaceutical organizations, according to the results of this study, focus on management support and user participation as organizational factors to develop a better perception among users regarding BDA.

Furthermore, all factors of the individual perception of BDA have significant relationships with job fit and profession fit except for image and compatibility with job fit and results demonstrability with profession fit. Previous studies’ results and the identity theory have already elaborated that relative advantage is the key predictor of job fit and profession fit [6, 58]. Another study concluded that relative advantage is the only driver of person-technology fit [51]. Therefore, the results of this study also show that relative advantage has a significant positive relationship with both job fit and profession fit. Pharmaceutical organizations should focus on all factors of innovation diffusion theory to create a sense of job and profession fit in employees but focuses on the relative advantage and complexity factors of BDA. The results of the study justified our proposal that both person-technology fit factors are a strong predictor of CRM capabilities, which ultimately enhance sales performance.

The findings of this research try to address the unique challenge of the salesforce by strengthening the relationship between the salesforce and customers through technology. Empirical analytics of data demonstrate that individual characteristics (self-efficacy, playfulness, and social norms) and organizational characteristics (voluntariness, user involvement, user participation, management support) along with innovation diffusion theory (IDT) factors (relative advantage, visibility, image, compatibility, complexity, and result demonstrability) are positive predictors of individual perception of BDA. This means that if pharmaceutical organizations want to adopt BDA in the organization, they should focus on individual, organizational, and IDT factors.

According to the results of this study, individual perception of BDA creates person-technology fit. This means that if the employees of the organizations feel that BDA has a relative advantage over other systems or technologies and if it has a good image in the mind of employees, better visibility, is more compatible with their job needs, decreases complexity and has better demonstrability of the results, then they feel more job fit and profession fit. Therefore, organizations that achieve a person-technology fit belief in employees should focus on these factors, especially relative advantage and complexity, because according to the results of this study and prior literature, these two are the most important constructs that influence person-technology fit [14]. Employees’ perception that the use of BDA can better fulfill their job and professional requirements will develop by achieving person-technology fit. The misfit of job and professional requirements with the system was the cause of SFA system failure [3, 6, 7].

The results of this study also provide evidence that job fit and profession fit have a positive influence on CRM capabilities and sales performance. This indicates that the salesforce perceives that BDA will facilitate a stronger relationship with customers and increase sales performance. Moreover, CRM capabilities also have a positive influence on sales performance. Therefore, pharmaceutical organizations should focus on individual, organizational and IDT factors to build positive perceptions of BDA, adopt BDA in organizations, and build job and profession fit perceptions in organizations, which will ultimately lead organizations to increase sales performance and CRM capabilities.

## Conclusion, implications, limitations, and future work

### Conclusion

Based on the study finding, organizations need to equipped their salesforce with BDA to strengthen their relationships with customers and sales. This study empirically offers a strong basis for further extending the research of BDA in sales and CRM capabilities. The study concluded that individual characteristics, organizational characteristics and innovation diffusion theory factors (relative advantage, visibility, image, compatibility, complexity, and results demonstrability) can build a positive perception of BDA in the mind of the salesforce. The positive perception of BDA will push the salesforce towards BDA acceptance in organizations. The study also highlighted that a positive perception of BDA increases the sense of job and profession fit in the mind of the salesforce. The study concluded that positive perception of BDA creates a person-technology fit belief in the salesforce, and this positive perception increases the sales performance and CRM capabilities of pharmaceutical organizations.

### Theoretical and practical implications

This study contributes greatly to theory and practice. First of all, the study extends the identity theory in the context of big data analytics. This study contributes by providing BDA as a substitute for failed SFA systems and provides a basis to extend the literature on the role of BDA in sales performance and CRM capabilities. This study contributes in theory by investigating a theory-driven framework that may be an important lens through which to examine BDA importance in sales performance and increments in CRM capabilities. Furthermore, this study investigated the combined effects of general technology acceptance theory factors and identity theory in the context of BDA which is lacking in the BDA literature. Moreover, the study also contributes to theory by extending innovation diffusion theory with the identity theory along with individual and organizational characteristics in the context of BDA. This study also enriches theory by exploring BDA in sales and CRM capabilities, which could be an important lens for technologically enhancing sales performance and CRM capabilities. Furthermore, this study provides the basis to further extend research regarding the role and impact of BDA in sales and CRM capabilities.

This study also practically contributes from many perspectives, similar to a theoretical contribution. First, this study provides an empirically tested model that helps in the adoption of BDA in pharmaceutical organizations. Furthermore, this study increases understanding in pharmaceutical organizations that the positive perception of the salesforce regarding BDA and person-technology fit are the most important factors in the acceptance of BDA and the resulting increase in their sales performance and CRM capability. This study also contributed practically in that it provides an alternative for organizations that are dissatisfied with the SFA system. The model provided an understanding of BDA implementation in particular and user acceptance of technology in general. Moreover, this study provides a basis for pharmaceutical organizations to start practicing BDA in sales and CRM to obtain the benefits of BDA and efficiently counter the challenges of sales and CRM capabilities.

### Limitations of the study and future work

In addition to the contributions of this study, we recognize some limitations, and some future directions of research are proposed on the basis of these limitations. First, the data gathered for this research from Pakistan and the results of this research might change in a cross-cultural context. Future research could pay more attention to a multicultural context to increase the generalizability of the model. Furthermore, future research should focus on longitudinal research to strengthen the results. Second, the data were collected from a developing country, and the results of this research may be different in developed countries. Therefore, in the future, researchers can test this model in developed countries and study this research model to compare different countries and increase its generalizability and effectiveness. Although the research results explain a high percentage of variance in sales performance and CRM capabilities, there are also some variables that might have an impact on these two dependent variables, such as role conflict and role ambiguity. In the future, research can test these two variables in addition to the given model. Furthermore, researchers can also include the moderating influence of demographical factors such as age, gender, income, and experience in this research model to strengthen it further. In addition, future research can be conducted after the implementation of BDA and then the post-implementation perception of BDA in the salesforce can be investigated. The future studies also study the important direct relationships pf individual characteristics, organizational characteristics and individual perception of BDA directly with the CRM capabilities and sales performance.

## Supporting information

S1 Appendix (DOCX)

S1 Data (XLSX)
