# Supplementary material for: Impact of big data analytics on sales performance in pharmaceutical organizations: The role of customer relationship management capabilities
Source: PLoS One. 2021 Apr 28;16(4):e0250229. doi: 10.1371/journal.pone.0250229 (PMC8081224; doi:10.1371/journal.pone.0250229)
Supplement: S1 Appendix — (DOCX) [file pone.0250229.s001.docx]

# S1 Appendix

**Table A1: Measurement items**

| **Variable** | **Questions** |
| --- | --- |
| **Self-efficacy** | **SE1:** I could complete the job using the BDA if there was no one around to tell me what to do. |
|  | **SE2:** I could complete the job using the BDA if I had only the user manuals for reference. |
|  | **SE3:** I could complete the job using the BDA if I had a lot of time to complete the job for which the BDA was provided. |
|  | **SE4:** I could complete the job using the BDA if I had seen someone else using it before trying it myself. |
|  | **SE5:** I could complete the job using the BDA if someone else had helped me get started. |
|  | **SE6:** I could complete the job using the BDA if I could call someone for help if I got stuck. |
|  | **SE7:** I could complete the job using the BDA if I had just the built-in help facility for assistance. |
|  | **SE8:** I could complete the job using the BDA if someone showed me how to do it first. |
| **Playfulness** | **P1:** I feel myself as Spontaneous when I interacting with computers. |
|  | **P2:** I feel myself as imaginative when I interacting with computers. |
|  | **P3:** I feel myself as Flexible when I interacting with computers. |
|  | **P4:** I feel myself as Creative when I interacting with computers. |
|  | **P5:** I feel myself as Playful when I interacting with computers. |
|  | **P6:** I feel myself as original when I interacting with computers. |
|  | **P7:** I feel myself as inventive when I interacting with computers. |
| **Social norms** | **SN1:** People whose opinions I value, prefer me to use a BDA in my work. |
|  | **SN2:** At work, my colleagues who are important to me think that I should use BDA. |
|  | **SN3:** At work, my superiors think that I should use BDA. |
|  | **SN4:** At work, my subordinates think that I should use a BDA. |
| **Voluntariness** | **VN1:** My superiors expect me to use the BDA. |
|  | **VN2:** My use of the BDA is voluntary. |
|  | **VN3:** Although it might be helpful, using the BDA is certainly not compulsory in my job. |
|  | **VN4:** My supervisor does not require me to use the BDA. |
| **User involvement** | **UI1:** I consider that the use of BDA to be relevant to me. |
|  | **UI2:** I consider that the use of BDA to be significant. |
|  | **UI3:** I consider that the use of BDA to mean a lot to me. |
| **User participation** | **UP1:** I played an important role in the design and/or development of the BDA. |
|  | **UP2:** I felt my opinion was adequately considered during the process of design and/or development of the BDA. |
|  | **UP3:** I participated in the design and/or development of the BDA. |
| **Management support** | **MS1:** The use of the BDA is encouraged by management. |
|  | **MS2:** My manager supports the use of the BDA. |
|  | **MS3:** My supervisor encourages the use of the BDA. |
| **Relative advantage** | **RA1:** Using the BDA enables me to accomplish tasks more quickly. |
|  | **RA2:** Using the BDA enhances my effectiveness on the job. |
|  | **RA3:** Using the BDA makes it easier to do my job. |
| **Visibility** | **V1:** I have seen what others do using the BDA. |
|  | **V2:** In my organization, one sees the BDA on many desks. |
|  | **V3:** The BDA is not very visible in my organization. |
| **Image** | **I1:** People in my organization who use the BDA have a high profile. |
|  | **I2:** Having the BDA is a status symbol in my organization. |
|  | **I3:** Using a BDA improves my image within the organization. |
| **Compatibility** | **C1:** I think that Using the BDA is compatible with all aspects of my work. |
|  | **C2:** I think that using the BDA fits well with the way I like to work. |
|  | **C3:** I think that Using the BDA fits into my work style. |
| **Complexity** | **CLX1:** Interacting with the BDA does not require a lot of my mental effort. |
|  | **CLX2:** My interaction with the BDA is clear and Understandable. |
|  | **CLX3:** I find the BDA to be easy to use. |
|  | **CLX4:** I find it easy to get the BDA to do what I want it to do. |
| **Results demonstrability** | **RD1:** I would have no difficulty telling others about the results of using the BDA. |
|  | **RD2:** The results of using the BDA are apparent to me. |
|  | **RD3:** I believe I could communicate to other the consequences of using a BDA. |
| **Job Fit** | **JF1:** The BDA can increase the quantity of output for the same amount of effort. |
|  | **JF2:** Using the BDA has no effect on the performance of my job. |
|  | **JF3:** Using the BDA decreases the time needed for my important job responsibilities. |
|  | **JF4:** Using the BDA significantly increases the quality of output of my job. |
|  | **JF5:** Using the BDA increases the effectiveness of performing job tasks. |
| **Professional Fit** | **PF1:** Using the BDA increases the level of challenge in my career. |
|  | **PF2:** Using the BDA increases the flexibility of changing jobs. |
|  | **PF3:** Using the BDA increases the amount of variety in my career. |
|  | **PF4:** Using the BDA increases the opportunity for more meaningful work. |
|  | **PF5:** Using the BDA increases the opportunity for preferred career assignments. |
|  | **PF6:** Using the BDA increases the opportunity to gain job security. |
| **CRM** | **CRM1:** Compared to your pervious system, you perceive that BDA will convert data to customer knowledge. |
|  | **CRM2:** BDA will provide better Customer information infrastructure. |
|  | **CRM3:** BDA will helpful in alignment of incentives, customer strategy, and structure. |
| **Sales performance** | **SP1:** Utilization of BDA improves my closing rates. |
|  | **SP2:** Utilization of BDA improves my customer retention. |
|  | **SP3:** Utilization of BDA enables me to analyze reasons for won. |

**Table A2:Pilot testing results**

| **Constructs** | **Items** | **Loadings** | **Cronbach’s Alpha** | **Constructs** | **Items** | **Loadings** | **Cronbach’s Alpha** |
| --- | --- | --- | --- | --- | --- | --- | --- |
| **Self- efficacy** | SE1 | 0.896 | 0.963 |  | RAD3 | 0.781 |  |
|  | SE2 | 0.702 |  | **Visibility** | V1 | 0.790 | 0.964 |
|  | SE3 | 0.881 |  |  | V2 | 0.757 |  |
|  | SE4 | 0.721 |  |  | V3 | 0.763 |  |
|  | SE5 | 0.749 |  | **Image** | I1 | 0.849 | 0.923 |
|  | SE6 | 0.880 |  |  | I2 | 0.881 |  |
|  | SE7 | 0.892 |  |  | I3 | 0.922 |  |
|  | SE8 | 0.785 |  | **Compatibility** | C1 | 0.851 | 0.909 |
| **Playfulness** | P1 | 0.716 | 0.969 |  | C2 | 0.879 |  |
|  | P2 | 0.887 |  |  | C3 | 0.921 |  |
|  | P3 | 0.870 |  | **Complexity** | CLX1 | 0.743 | 0.969 |
|  | P4 | 0.901 |  |  | CLX2 | 0.731 |  |
|  | P5 | 0.884 |  |  | CLX3 | 0.756 |  |
|  | P6 | 0.915 |  |  | CLX4 | 0.791 |  |
|  | P7 | 0.737 |  | **Results demonstrability** | RD1 | 0.754 | 0.854 |
| **Social Norms** | SN1 | 0.805 | 0.961 |  | RD2 | 0.726 |  |
|  | SN2 | 0.817 |  |  | RD3 | 0.714 |  |
|  | SN3 | 0.781 |  | **Job Fit** | JF1 | 0.811 | 0.912 |
|  | SN4 | 0.819 |  |  | JF2 | 0.814 |  |
| **Voluntariness** | VN1 | 0.760 | 0.905 |  | JF3 | 0.781 |  |
|  | VN2 | 0.772 |  |  | JF4 | 0.877 |  |
|  | VN3 | 0.828 |  |  | JF5 | 0.855 |  |
|  | VN4 | 0.716 |  | **Professional Fit** | PF1 | 0.703 | 0.913 |
| **User involvement** | UI1 | 0.754 | 0.867 |  | PF2 | 0.737 |  |
|  | UI2 | 0.789 |  |  | PF3 | 0.734 |  |
|  | UI3 | 0.711 |  |  | PF4 | 0.709 |  |
| **User participation** | UP1 | 0.707 | 0.852 |  | PF5 | 0.711 |  |
|  | UP2 | 0.768 |  |  | PF6 | 0.763 |  |
|  | UP3 | 0.740 |  | **CRM capabilities** | CRM1 | 0.806 | 0.879 |
| **Management support** | MS1 | 0.773 | 0.865 |  | CRM2 | 0.732 |  |
|  | MS2 | 0.737 |  |  | CRM3 | 0.761 |  |
|  | MS3 | 0.764 |  | **Sales Performance** | SP1 | 0.749 | 0.836 |
| **Relative advantage** | RAD1 | 0.795 | 0.788 |  | SP2 | 0.799 |  |
|  | RAD2 | 0.746 |  |  | SP3 | 0.768 |  |
